# Global and Local Trends Affecting the Experience of US and UK Healthcare Professionals during COVID-19: Twitter Text Analysis

**DOI:** 10.3390/ijerph19116895

**Published:** 2022-06-04

**Authors:** Ortal Slobodin, Ilia Plochotnikov, Idan-Chaim Cohen, Aviad Elyashar, Odeya Cohen, Rami Puzis

**Affiliations:** 1 Department of Education, Ben-Gurion University of the Negev, Beer-Sheva 8410501, Israel; ortalslo@bgu.ac.il; 2 Department of Software and Information Systems Engineering, Ben-Gurion University of the Negev, Beer-Sheva 8410501, Israel; iliapl@post.bgu.ac.il; 3 Cyber@BGU, Ben-Gurion University of the Negev, Beer-Sheva 8410501, Israel; 4 School of Public Health, Faculty of Health Sciences, Ben-Gurion University of the Negev, Beer-Sheva 8410501, Israel; idanchai@post.bgu.ac.il; 5 Department of Computer Science, Sami Shamoon College of Engineering, Beer-Sheva 8410802, Israel; aviadel2@sce.ac.il; 6 Department of Nursing, Recanati School for Community Health Professions, Faculty of Health Sciences, Ben-Gurion University of the Negev, Beer-Sheva 8410501, Israel

**Keywords:** COVID-19, emotions, health and politics, healthcare professionals, twitter analysis, natural language processing

## Abstract

Background: Healthcare professionals (HCPs) are on the frontline of fighting the COVID-19 pandemic. Recent reports have indicated that, in addition to facing an increased risk of being infected by the virus, HCPs face an increased risk of suffering from emotional difficulties associated with the pandemic. Therefore, understanding HCPs’ experiences and emotional displays during emergencies is a critical aspect of increasing the surge capacity of communities and nations. Methods: In this study, we analyzed posts published by HCPs on Twitter to infer the content of discourse and emotions of the HCPs in the United States (US) and United Kingdom (UK), before and during the COVID-19 pandemic. The tweets of 25,207 users were analyzed using natural language processing (NLP). Results: Our results indicate that HCPs in the two countries experienced common health, social, and political issues related to the pandemic, reflected in their discussion topics, sentiments, and emotional display. However, the experiences of HCPs in the two countries are also subject to local socio-political trends, as well as cultural norms regarding emotional display. Conclusions: Our results support the potential of utilizing Twitter discourse to monitor and predict public health responses in emergencies.

## 1. Introduction

Healthcare professionals (HCPs) are an essential public health resource for improving health outcomes during routine and emergencies [1,2]. As is the case in many natural and human-made disasters, HCPs are at the frontline in combatting the medical and social effects of the COVID-19 pandemic [3]. Studies have shown that HCPs were at increased risk for being infected with COVID-19 (15–20% of the infected population). Emergency clinicians and specialties with high exposure to aerosol-generating procedures were especially vulnerable [4,5]. In addition, recent reports have indicated that HCPs were at increased risk for emotional difficulties associated with the pandemic such as fear of death, feelings of loneliness, and depression, with high prevalence among females, nurses, and frontline responders [6], for meta-analysis.

HCPs have a far-reaching influence on health system leaders, individuals, and families when it comes to disaster preparedness, response, and recovery [7]. According to the WHO [1], the HCP workforce advances sustainable development, with respect to increasing the quality of education, reducing poverty, providing decent work conditions, increasing inclusive economic growth, and reducing gender inequality. HCPs have rich population-based knowledge, skills, and expertise, and they can be found in diverse professional and community settings, collaborating with a wide range of HCPs. Thus, understanding HCPs’ emotional display during emergencies is a critical aspect of increasing the surge capacity of communities and nations.

### 1.1. Healthcare Professionals and Social Media

Social media has been recognized as a dimension of healthcare and a mechanism of social interaction for engaging patients, HCPs, and the healthcare systems [8]. Twitter was found to be the most popular form of social media used for healthcare communication [9]. According to Stukus [10], online conversation among patients is already happening, but the professional voice of HCPs is lacking. Studies that measured HCPs’ involvement in professional conversations about health during the COVID-19 pandemic found their involvement to be insufficient. For example, Herrera-Peco et al. [11] observed very limited participation of HCPs in the dissemination and generation of information led by the Spanish Ministry of Health. In view of the central role of social media in public discussion during the COVID-19 pandemic [12], it is important to understand whether and how HCPs are emotionally and professionally involved in public discourse during this period.

### 1.2. Emotions of Healthcare Professionals

Although HCPs often have to deal with strong, unexpected emotions arising from both their patients and themselves, the expression of emotions by HCPs has traditionally been considered unprofessional and inappropriate, and basically a sort of “taboo” [13]. However, the study of emotions is fundamental to the practice of healthcare. Previous research has shown that HCPs’ emotional state plays a critical role in their response to situations, patients, and colleagues, and plays an intrinsic role in clinical judgment, patient safety, and communication [14]. Emotions are important for building mutual trust, processing information, and even determining people’s health choices [15].

Emotions are subjective reactions to given environmental events, whether internal or external, and are characterized by physiological, cognitive, experiential, and behavioral changes. Such changes allow individuals to attribute meaning to their experiences and prepare for given actions [16].

Psychologist Paul Ekman [17] identified six basic, universal emotions. These emotions were happiness (joy), sadness, disgust, fear, surprise, and anger. Studies have found that emotional regulation and disclosure among HCPs may vary based on cultural and situational contexts [18,19]. Rakovski and Price-Glynn [18] provided some evidence that the experience of stress and the exertion of emotional labor among nurses may differ between cultures. They found that noncitizen nurses were more satisfied with their jobs and careers than citizen nurses. In addition, among nurses with American citizenship, minority groups had the greatest levels of job and career satisfaction. These findings suggest that emotional labor can vary depending on the cultural context and encouraged us to examine how nurses in different countries respond to ongoing emotional demands.

### 1.3. Emotional Experiences of HCPs during Emergency

The emotional experience and expression of healthcare professionals are even more vital in emergency situations, when providers must work under extreme medical and emotional demands, often during short periods. Healthcare workers arguably experience elevated anxiety and are predisposed to the greater negative psycho-social impact of the current COVID-19 pandemic. Frontline HCPs are exposed to hazards that include pathogen exposure, long working hours, burnout, loneliness, fatigue, disorders of mental health (e.g., fear, anxiety, depression), occupational stigma, and physical and psychological violence with a potential negative impact on patient safety and occupational health [20,21].

A recent analysis of more than 50,000 Twitter profiles of HCPs during the COVID-19 [22] showed how HCPs’ emotional displays and topics of interest resonated with local and global events. This study showed that, overall, during 2020 HCPs experienced more negative emotions and fewer positive emotions, compared with the year before. Moreover, this study underscored the key role of HCPs in anticipating, maintaining, and affecting social and economic changes during an emergency. To take a step forward, the current study examined to what extent HCPs’ discourse and emotional display are associated with local social, cultural, and political contexts.

### 1.4. The Current Study

Healthcare in the United Kingdom and the United States has been at the center of political, social, and cultural debate in the past few years. On 24 October 2019—45 days before the world’s first suspected case of COVID-19 was announced—the Global Health Security Index ranked the American and the British healthcare systems in the first and second place (out of 195 countries) among countries that were most prepared to tackle a serious outbreak. Nevertheless, in June 2020, the US and UK ranked first and second, respectively, in the number of deaths, due to the COVID-19 [23].

Despite the similar magnitude of the COVID-19 pandemic in these two socio-economically favorable countries (that might be partly associated with the political context in which a national policy response to a pandemic is formulated and implemented), their healthcare systems greatly differ in terms of cost, quality, workforce, and access. The US healthcare system is predominately made up of private services providers and is considered the most expensive system, compared with other industrialized or OECD countries. In contrast, the UK healthcare system is largely universal, whereby healthcare is perceived as an entitlement and a human right [24]. Since the UK healthcare system is largely funded by the central and devolved governments, it is the largest employer in the UK [25]. Compared with the UK, the quality of the US healthcare system is largely considered inefficient, due to heavy annual spending, the absence of universal healthcare, and poor accessibility in the midst of escalating healthcare costs [26,27]. It is, therefore, important to examine whether and how these macro-level economic, political, and institutional differences are translated into HCPs’ experiences during both routine times and emergencies.

In this research, we studied the discourse of English-speaking HCPs on Twitter and basic emotions reflected in their tweets, using natural language processing (NLP). We put special focus on the differences and similarities between US and UK before and during the COVID-19 pandemic (January 2019–December 2020).

## 2. Materials and Methods

In this study, we analyzed public tweets published by HCPs from 1 January 2019 to 6 December 2020.

### 2.1. Population

To identify the HCP population, we first identified a list of healthcare points of interest (POIs) and collected their followers. Then, we applied active machine learning to mark accounts of individual HCPs in this large bulk of followers. The detailed process is described in [22]. The current study used a semi-bounded data collection strategy that focuses on a defined set of Twitter users (i.e., HCPs), without restrictions on the topical content. This method may yield either full coverage of the user accounts of interest or a close approximation of all tweets of interest and currently informs the bulk of substantive research using Twitter [28].

Identifying HCP POIs—We defined one list of general 16 health professions and 23 specializations recognized by the American Board of Medical Specialties (see the full list in Table A1). In parallel, we defined a second list containing types of POIs, such as conferences, unions, and journals. Then, we created short keyword queries by taking one keyword from each of the two lists. We searched Twitter using the short keyword queries in order to retrieve accounts of the HCP POIs. The results were manually inspected to identify accounts managed by HCP POIs such as facilities, organizations, and venues related to healthcare resulting in 522 POIs. Prioritizing those that follow multiple POIs. In this study, we analyzed tweets that originated in the US or the UK, based on indications in the Twitter accounts.

Inclusion/Exclusion Criteria—Among the followers of the HCP POIs, many are accounts of organizations rather than individuals or accounts of patients and professions not directly related to healthcare. We define an HCP as an individual working in the healthcare system or a student studying a medical profession. Students were included since they typically go through hands-on training that involves interacting with patients. We excluded therapists that work in fields that are generally considered complementary or alternative medicine and art therapy. To achieve the criteria of HCPs, we trained two support vector machine (SVM) classifiers to filter out organizational and non-HCP Twitter accounts based on term frequency–inverse document frequency (TF–IDF) features extracted from the accounts’ description and full names. To reduce the manual labeling effort, we used active learning with an uncertainty sampling strategy. In this strategy, the human annotators manually inspect and label accounts that the classifier is least certain about.

Ethics—Texts with identifiers were stored on a secure server at the Ben-Gurion University of the Negev. After completing the data analysis, the contents of the posts were deleted. This method of deleting Twitter content and storing identifiers (without account holder information) is common in such studies and is in accordance with Twitter’s user license agreement.

### 2.2. Data Analysis

The data analysis consists of four steps. Standard text preprocessing techniques were applied in the first step. Due to the informativeness of hashtags on Twitter, they were not removed, although the hashtag symbol (#) was removed. In addition, we removed terms signifying the COVID-19 disease or the SARS-CoV-2 virus. In the second step, topic models were obtained using the latent Dirichlet allocation (LDA) algorithm [29] implemented in the Gensim 3.8.3 library [30].

The dataset was partitioned into 20 topics where every tweet was associated with a single topic. We manually inspected the top 50 words within each automatically generated topic, as well as the contents of a few hundred tweets with the highest probability of belonging to the topic. Based on this manual inspection, the nine most coherent topics were selected. For each topic, the weekly number of tweets during 2020 was tracked. This allowed us to identify the major volume changes for these topics and associate them with significant world events, which presumably corresponded to the volume changes.

To examine the relationships between topics, we calculated the conditional probability P(t∈A |t∈B) of a random tweet t belonging to topic A given that it belongs to topic B for each pair A-B of topics. In order to perform this calculation, we relied on the tweet-topic probabilities computed by the LDA algorithm. The conditional probability P(t∈A |t∈B) was averaged over all tweets originating from the UK and over all tweets originating from the US to produce the directed relationship P(A |B) between all pairs of topics in each country. We focused specifically on the topic of public health and social values and their decomposition to other subtopics.

In the third step, to estimate the sentiments expressed by HCPs for each topic, the Valence Aware Dictionary and sEntiment Reasoner (VADER) was used, which is a lexicon and rule-based sentiment analysis tool [31]. We used a pretrained recurrent neural network model developed by Colneric and Demsar [32] to quantify the probabilities of Ekman’s six basic emotions [17] expressed in the text. For each emotion, the annual and weekly average emotion levels were calculated. Differences between average emotion levels among countries and between 2019 and 2020 were examined based on the emotion distribution using Welch’s *t*-test for normally distributed emotions (anger, sadness, and joy) and the Mann–Whitney U test for non-normally distributed emotions (fear, surprise, and disgust).

**Figure 1:**
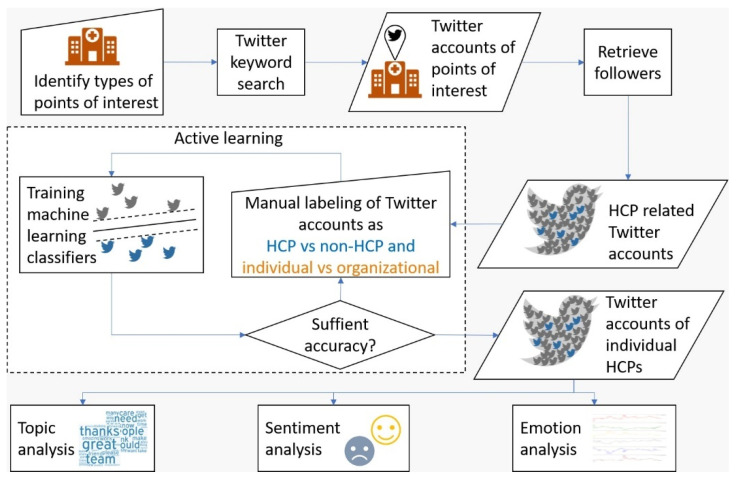
Steps of study methodology.

Finally, in the fourth step, we analyzed the time-course of each emotion during 2020 (47 weeks) and quantified their correlation with the number of new COVID-19 cases (Confirmed), the number of deaths caused by COVID-19 (Deaths), their weekly change rates (∆Confirmed and ∆Deaths, respectively), and the estimated reproduction rate of SARS-CoV-2 (*R_t_*). A Shapiro–Wilk test was conducted to examine variables’ distribution. Cross-correlation analysis was performed to account for possible lags at a range of 1–8 weeks between the pandemic development and emotional response. Figure 1 displays the steps involved in the study’s methodology.

## 3. Results

### 3.1. Study Population

The study population consists of 25,207 tweet authors, of whom 90% are expected to be individual HCPs based on our manual validation of a random sample. The study was conducted throughout 2020, on tweets that originated in the US or UK. In 2020, the authors posted around 4.5 M tweets. The characteristics of the authors collected from Twitter, by country of residence, are presented in Table 1.

### 3.2. Content of Discourse

The nine topics constitute 95% of the total discourse of the US authors and 96.6% of the UK authors in our study population (see Table 2). The tweets on these topics discuss the following issues: (1) public health and social values—public health policy, its applications, and social values mainly related to aspects of health; (2) day-to-day life—everyday situations; (3) food—food-related matters; (4) politics—politics and government; (5) professional achievements—comments, as teams and individuals, on professional accomplishments; (6) medical studies and COVID-19 information—medical studies and epidemiological information related to COVID-19; (7) loss and consolation—empathy and consolation to individuals and families on their losses; (8) account promotion—information about accounts’ Twitter activity; and (9) picture challenges—an online challenge prompting Twitter users to post pictures representing their lives.

The tweets in the identified topics discuss professional-related issues such as public health and social values (47.2% and 42.8% for the UK and US HCPs, respectively) and non-professional issues such as day-to-day life and food. The topics, prevalence, and average sentiment score are presented in Table 2. For both US and UK HCPs, 26% of their discourse was focused on public health and social values, and about 1% of their discourse was devoted to loss and consolidation. However, while UK HCPs were more occupied with daily life (32.6% and 24.8% for the UK and US HCPs, respectively) and professional achievements (14.2% and 8.4% for the UK and US HCPs, respectively) than US HCPs, their US peers were more engaged in political issues (8% and 3.2% for the US and UK HCPs, respectively) and discussions on food (15.5% and 10.3% for the US and UK HCPs, respectively). Examining the sentiment scores revealed significant differences among the sentiment scores for all topics, with UK authors presenting significantly higher sentiment throughout the discourse. The sentiment scores for the political topics were found to be the lowest for authors from both countries. However, the greatest difference between the US and UK authors was seen in the political topic, with the ratio between the UK and US authors being 3.7, indicating a more positive attitude toward politics among UK authors.

While Table 2 presents the general distribution of topics discussed by HCPs during 2020, Table 3 focuses on the discussions related to politics. In both countries, 15–22% of tweets related to politics were also related to other topics (the topics of public health and social values and day-to-day life). Despite being a significant fraction of the discourse on politics, these two topics are less emphasized in the context of politics than within the general unconditional discussions. The probabilities of other topics within the topic of politics are roughly the same as their a priori probabilities.

### 3.3. Discourse Trends in 2020

**Figure 2:**
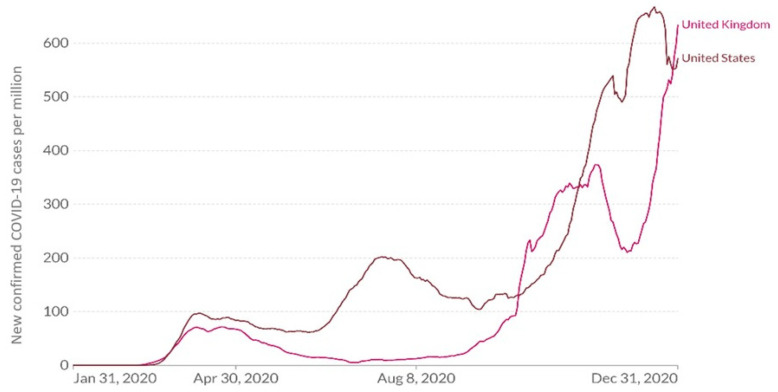
Daily new confirmed COVID-19 cases (per million) in the US and UK in 2020 [33].

The daily number of new confirmed COVID-19 cases (per million) in the US and UK during the study period is presented in Figure 2. The differences in COVID-19 development in the two countries are illustrated; the first wave of the pandemic began in the US and UK simultaneously, while the second and the third waves began earlier in the US.

**Figure 3:**
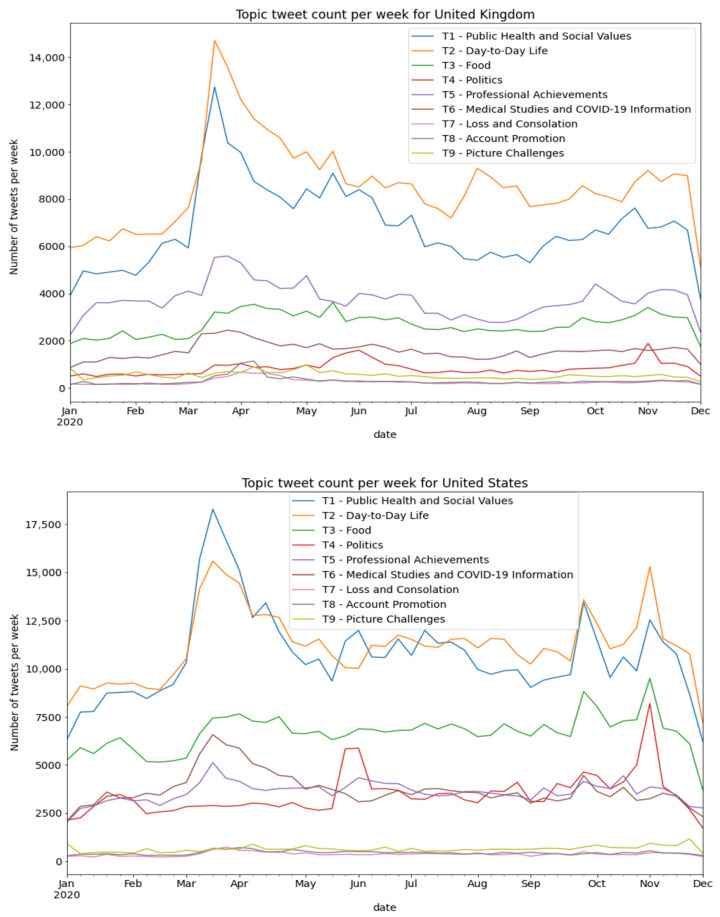
Discourse trends over time—number of tweets per week by US and UK HCPs in 2020.

Figure 3 presents the number of tweets on the nine identified topics over the course of the year (2020). The volume of the discourse of both US and UK HCPs increased at the beginning of the COVID-19 pandemic. As seen in the figure, in both countries, two topics—daily life and public health and social values—were the most prevalent. However, among the US authors, the topics of daily life and public health, and social values were equally prevalent, while in the UK, the discussion of daily life was the most prevalent throughout the year. The topic of food maintained its prevalence throughout the year, with a greater volume of discourse seen by the US authors. Politics was discussed by both US and UK authors, with an increase in volume (a sharp increase in the discourse of US authors and a slight increase among UK authors) following George Floyd’s death and around the American elections. Spikes in the overall number of tweets of US authors around 30 September, 23 October, and 3 November might be reflective of the presidential debates that occurred on those dates.

### 3.4. Emotion Analyses among US and UK HCPs

**Figure 4:**
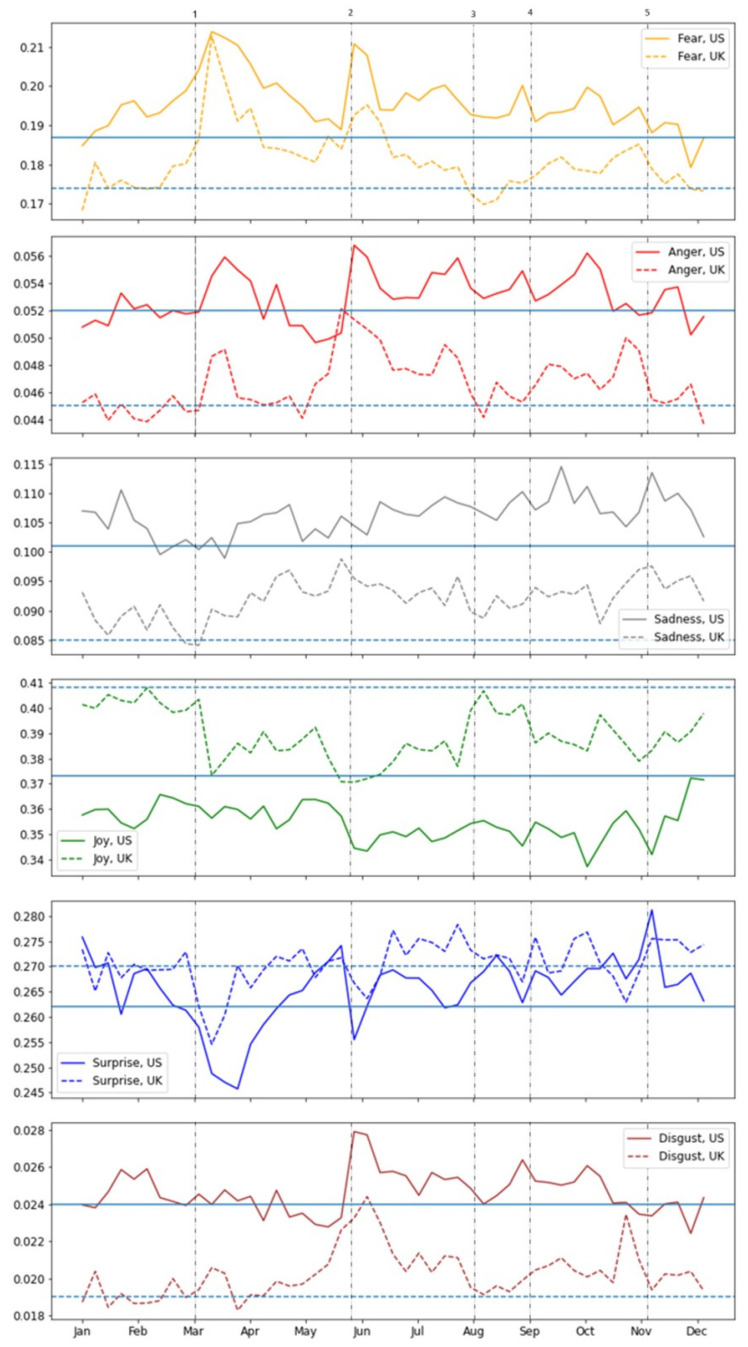
Average weekly emotion values in 2020. Note: the horizontal lines represent the mean score for each country in 2019 (solid—US, dashed—UK). 1—first wave of COVID-19 in the UK and the US; 2—the George Floyd incident; 3—second wave of COVID-19 in the US; 4—second wave of COVID-19 in the UK; 5—US elections.

Emotion analyses were conducted for the six basic emotions—anger, joy, fear, sadness, surprise, and disgust, as proposed by Ekman (1992). The mean values of HCPs’ emotions in the US and UK before the pandemic (in 2019) and during the pandemic (2020) are compared in Table A2. Significant differences were found between all values in accordance with the country—namely, US authors had higher scores than UK authors for the negative emotions (fear, anger, sadness, and disgust) and lower scores for the positive emotions (joy and surprise). The differences between the emotional mean values before and during the pandemic among US and UK authors are presented in Table A3. For both US and UK authors, there were significant differences seen over the years, i.e., negative emotions (fear, anger, sadness, and disgust) increased significantly during 2020, and joy decreased significantly in 2020 among authors in both countries. Figure 4 shows that the major trends seen are shared by both US and UK authors, with negative emotions increasing during most of the pandemic period and a corresponding decrease in positive emotions. During the first pandemic wave (similarly in the US and UK), there was a sharp increase in fear, which gradually became moderate during the pandemic timeline, together with an increase in sadness that appeared after the first wave of the pandemic. The effect of George Floyd’s death and the antiracism protests extended beyond the boundaries of the US and was expressed in the emotions of UK authors—a sharp increase in fear and anger—with the first increase in disgust seen for these HCPs.

The significant correlations of emotions with the development of the pandemic are summarized in Table 4, which presents the correlations between fear and anger, the virus reproduction rate, and ∆Deaths, together with the correlation significance and the identified lags. Both US and UK authors expressed fear following the increase in the virus reproduction rate and expressed anger in the face of death.

## 4. Discussion

Previous studies have shown that the role of HCPs in emergencies exceeds far beyond providing medical care and that they have a significant impact on the social and economic functioning of communities during disasters [1]. While HCPs worldwide have been praised for their frontline efforts in the care and treatment of patients with COVID-19, very little is known about how HCPs have responded to the current emergency and the specific global and local events that trigger their responses [34]. Using an analysis of HCPs’ Twitter posts before and during the COVID-19 outbreak in the US and UK, the current study examined to what extent HCPs’ discourse and emotional display are associated with local and global social, cultural, and political contexts.

Overall, our results indicate that HCPs in the US and UK faced similar health and social challenges related to the pandemic, which is reflected in their discussion topics, sentiments, and emotional display. However, the experiences of HCPs in the two countries are also subject to local socio-political trends, as well as cultural norms in their emotional display. Some key findings and lessons that emerge from our results are summarized below.

### 4.1. Discourse Topics and Sentiments

Our analysis of the discussion topics highlighted similarities and differences between the experience of HCPs in the US and the UK. For HCPs in both countries, the greatest proportion of discussion was directly associated with professional content (public health, social values, personal achievement, and COVID-19). In both countries, a full quarter of the discussions were related to public health and social values, which was the leading topic of interest. In both countries, just 1% of HCPs’ discussions focused on loss and condolences. However, British HCPs dedicated a larger proportion of their discussions to daily life issues and professional achievements, while American HCPs were significantly busier with discussions on politics and food.

We expected that HCPs would share their impressions of (or reflections on) their daily encounters with illness and death on social media. However, in contrast to our expectations, loss and trauma were underrepresented in HCPs’ discussions. This finding aligns with the recent work of Ojo et al. [35], who found that tweets with healthcare-led hashtags expressed more positivity and more action-oriented language than non-healthcare-initiated hashtags. It is possible that the reluctance of HCPs to share negative events on social media reflects societal expectations of professionalism from medical experts [13,36].

Similarities and differences between the two groups (US and UK) were also pronounced in the sentiments that accompanied their discussions. Both US and UK authors expressed fear after the virus reproduction rate increased and anger in the face of death. Additionally, in both the American and British groups, discussing politics was associated with the lowest level of positive sentiments. However, we found that British HCPs were more likely to express positive sentiment than American HCPs across all discussion topics.

Examining time-related changes in the topics discussed showed an increase in the number of posts published by the two groups at the beginning of the pandemic. Moreover, political discourse increased in both groups following George Floyd’s death (25 May 2020) and the American elections (3 November 2020), with a sharp increase seen in the discourse of HCPs from the US and a slight increase among HCPs from the UK. Overall, however, the groups differed in terms of the trends in the topics discussed. For example, American HCPs published an increasing number of posts on public health and social values as the pandemic waves approached their peaks, whereas in British HCPs such a trend was obtained only in the first wave.

### 4.2. Emotional Display

Consistent with previous studies on the general population [37] and HCPs [38], among both British and American HCPs, we observed an increase in negative emotions and a decrease in positive emotions throughout 2020 (compared to 2019). For instance, a recent study examining the expression of the six basic emotions on Twitter during the COVID-19 pandemic in Portugal revealed an association between the emotional patterns observed in tweets and the pandemic’s evolution, with more negative tweets posted at the beginning of the pandemic [39].

As is evidenced by prior studies [20,21], HCPs in both counties shared a sharp increase in their levels of fear during the first wave of the pandemic, which gradually reduced with time, and an increase in sadness that was maintained over time. Fear was also experienced by both groups in response to an increase in the virus reproduction rates, whereas anger was experienced by both groups in response to a rise in the number of COVID deaths. Despite the similar patterns of emotional response, the magnitude of HCPs’ emotions was higher among American HCPs than among their British counterparts.

Interestingly, our results showed that HCPs from both the US and the UK responded in a similar way, emotionally, to global health changes as well as to socio-political events. Our analysis showed that the death of Gorge Floyd was reflected by a sharp increase in fear, anger, and disgust among American, as well as British HCPs. As in the US, this event also resulted in a wave of protests in the UK, which had the largest Black Lives Matter protests in the world outside the US. Even before George Floyd’s death, protesters were already galvanized by the death of a Black transport worker in London. The worker was killed in a racist attack at work that occurred after she reported that a White man spat on her and was denied proper protection [40].

Together, these results paint a detailed and dynamic portrait of HCPs’ state of mind in times of emergency [22,34]. Specifically, these findings suggest that HCPs around the globe are influenced by similar health, social, and political events but that these events elicit a similar profile of cognitive and emotional responses. For example, our results demonstrated that both British and American HCPs experienced elevated levels of negative emotions with increasing number of COVID-19 case, as well as with political instability. These results are in line with previous studies showing that changes in mood word frequencies on Twitter corresponded with real-world events, such as the unexpected deaths of popular individuals, public unrest, or natural disasters [41,42]. Such studies showed that negative mood indicators coincided both with periodic events, such as holidays or the new school year, but also with major socio-political events, such as the announcement of public spending cuts by the government and riots [43,44]. Thus, this study provides further support for the vast potential of utilizing Twitter to monitor and predict public health responses in times of emergency [45,46].

In addition to the similarities observed in the quality and timing of American and British HCPs’ responses to the pandemic, our findings revealed important differences between the two groups. These differences were found in the discussion topics, as well as in the prevalence and magnitude of emotional expression. Generally, HCPs in the UK reported fewer and weaker negative sentiments and emotions than their counterparts in the US, both before and during the COVID-19 outbreak.

Several explanations may shed light on the consistent differences observed between American and British HCPs in the prevalence and magnitude of the expression of negative emotions. One possible explanation relates to cultural differences regarding the rules of emotional display. Although the US and UK share a common language and a historical past, they often differ in the socio-cultural norms of emotional expression [47,48]. Previous studies have suggested that Americans are more likely to express negative emotions (particularly at work) than people from Britain. These studies suggest that the British are more driven by social desirability and have a greater reluctance in disclosing their feelings or expressing their emotions [45]. Moreover, a study examining the emotional expression of workers from the US and UK found that American workers were more likely than British workers to expect warmth from their colleagues and more likely to expect this to be genuine than the British. Additionally, British workers were significantly more likely to suppress their anger in their workplace than Americans [48].

Another possible explanation for the differences observed between the groups relates to differences in language expression of emotions. Based on an analysis of words carrying emotional content in 20th-century English language books, Acerbi et al. [49] found that American English has become decidedly more “emotional” than British English. This difference has apparently developed only since the 1960s as part of a more general stylistic differentiation between American and British English. The authors suggested that the relative increase in American mood words was associated with both the increase in antisocial and narcissistic sentiments in American popular culture and a corresponding decrease in words indicating social interactions. While these findings do not indicate that Americans are more emotion-driven in their real-world interactions, their use of words is an informative sample of possible cultural differences.

From another point of view, the differences observed between the US and UK HCPs may be associated with the healthcare systems’ response to the COVID-19 crisis [24,25,26,27]. While all countries faced common challenges, including a shortage of medical technology and scaling up testing capacity [50], differences exist in health system capacity and political leadership. A recent comparative analysis of health policy responses to COVID-19 in Canada, Ireland, the UK, and the US from January to November 2020 suggested that in the US, the lack of universal health coverage has created barriers to accessing care and political pushback against scientific leadership, thus negatively affecting crisis response [51].

### 4.3. Relationships between Politics and Health

Relationships between politics and public health have been discussed in the literature; for example, one study examined the impact of government styles and health outcomes over the years [52]. However, the COVID-19 pandemic accelerated and intensified political aspects of the health process [53]. The close connection between politics and pandemics is a known issue. According to Tognotti [54], pandemics’ control has always been controversial, since the associated policies raise ethical, political, and socioeconomic issues, trying to balance public interest and individual rights.

Next, we discuss how this study highlights the political aspects of HCPs’ discourse. First, we found that the public health and social values topics had the second-highest relationship with the political topics (after day-to-day life, which had the greatest relationship), as seen in Table 3. Second, the political topic maintains its volume throughout 2020 among US and UK HCPs, with increases seen around the Gorge Floyd incident and the American election. While of all the topics examined, sentiment for the political topics was found to be the lowest in both countries, UK authors were more positive and spoke about political issues less frequently than the US authors. Sentiment in political discourse is sometimes used as a proxy for trust [55]. Thus, the low sentiment score of the political topic in both countries could be an indication of the low level of trust that HCPs have in politicians and the policies they promote. The issue of trust is crucial during emergencies for increasing citizens’ adherence to guidelines, especially in the health context [56]. The WHO [57] defined risk communication as “the real-time exchange of information, advice, and opinions between experts or officials and people who face a threat (hazard) to their survival, health, or economic or social wellbeing”. Its ultimate purpose is to enable everyone at risk to make informed decisions to mitigate the effects of the threat (hazard), such as a disease outbreak, and take protective and preventive action”. According to a recent report, the WHO [58] emphasized that risk communication has shifted from a focus on the dissemination of information to an improved understanding of the communication process leading to changes in behaviors and beliefs. To achieve this goal, and based on the results presented in this study, more attention should be given to the relationships between policymaking, politicians, and HCPs.

### 4.4. Limitations and Recommendations for Further Studies

There are a few limitations of this research that need to be considered. First, this study is limited to HCPs who are active on Twitter, and therefore, we cannot generalize our findings to the entire HCP population. In addition, since the topics, sentiment, and emotion analysis were performed using machine learning algorithms, our results are an estimation of HCPs’ experiences as opposed to experiences confirmed by HCPs. Finally, our data collection consists of tweets from January 2019 through January 2021, and additional waves of the COVID-19 pandemic have occurred since then. Further research on the long-term effects of the pandemic on the well-being of HCPs is needed.

## 5. Conclusions

Our findings highlighted the importance of HCPs’ voices in monitoring and predicting public health changes in times of crisis. Comparing the expression of sentiments and discussion topics of HCPs in the US and the UK showed that albeit facing similar global challenges, HCPs’ experiences were also influenced by cultural norms of emotional display and by local socio-political events. Our results should be considered under several limitations, including self-selection bias, the inclusion of two countries only, and the lack of information about HCPs’ responses to the pandemic after January 2021. These limitations highlight the importance of evaluating the long-term effects of the pandemic on HCPs. In addition to the well-known adverse effects of the COVID-19 on HCPs, future studies should also address positive changes and post-traumatic growth. For example, Feingold et al. [59], who studied HCPs’ responses following the first two waves of the COVID-19, found that 76.8% of participants endorsed moderate or greater post-traumatic growth. The most prevalent domains of growth were increased appreciation of life, improved relationships, and greater personal strength. Post-traumatic growth was negatively associated with pandemic-related PTSD symptoms and burnout. Additionally, it is necessary for future research to enrich our understanding of HCPs’ well-being in times of crisis in different socio-cultural contexts. Special attention should be given to HCPs in low-resource settings, such as communities with low economic status and culturally/linguistically diverse populations, who are at increased risk for suffering from the physical and social adverse effects of the pandemic [60].

The current study may contribute to practice and research in several ways. First, HCPs’ frequent use of social media suggests that this platform could also be used as a means of education and for distributing information [61]. Healthcare professionals are considered by the population as an essential element for the understanding of health-related messages, and their absence in social networks as reference elements may generate distrust and even disaffection toward truthful healthcare information [11]. As advocated by the WHO [58] in a recent strategic report, social media accounts (Twitter, Facebook) could be used for monitoring and provisioning training and educational materials for healthcare professionals and the evaluation of action plans.

Second, our results showed that negative mood indicators among HCPs coincided with COVID-19 case acceleration as well as with socio-political events. These results suggest that examining HCPs’ responses on social media could help policymakers better understand HCPs’ concerns regarding the COVID-19 pandemic. Third, the frequent use of social media by HCPs suggests that this platform provides an important means of communication in times of emergency and physical distancing. Previous reviews supported the use of social media for health interventions, health campaigns, medical education, disease outbreak surveillance, behavior change, and facilitating offline health-related services and events [62,63,64]. Moreover, the potential of social media such as Twitter in capturing pandemic-related sentiment may ultimately inform novel surveillance methods of individual emotional states and their spread [65].

Insights derived from social media monitoring could be applied to guide health organizations in the development of individual and group psychosocial interventions using social media platforms [66,67]. Finally, our results emphasize the close relationship between social and political trends and the resilience of the healthcare system. These findings should encourage policymakers and politicians to invest efforts in fostering trust between HCPs and the policymakers, which, in turn, would increase public trust in policymakers and politicians and improve risk communication.

## Figures and Tables

**Table 1:** The characteristics of the study population across the study period (2019–2020).

Statistics	US	UK
Number of accounts	14,113	11,094
Number of tweets	5,091,519	3,044,787
Average number of tweets	361	274
Average number of friends	626	515
Average number of followers	638	486
Total tweets published in 2019	2,203,328	1,381,416
Total tweets published in 2020	2,888,191	1,663,371

**Table 2:** Topic of discourse, prevalence, coherence, and average sentiment score in 2020.

Topic	Prevalence		Sentiment Score					
	US	UK	US			UK		
				95% CI			95% CI	
			Mean	Lower	Upper	Mean	Lower	Upper
Public health and social values	26.1%	26%	0.1	0.098	0.101	0.159	0.158	0.161
Day-to-day life	24.8%	32.6%	0.238	0.237	0.24	0.338	0.336	0.339
Food	15.5%	10.3%	0.056	0.055	0.058	0.189	0.186	0.192
Politics	8%	3.2%	0.023	0.02	0.025	0.086	0.082	0.091
Professional achievements	8.4%	14.2%	0.533	0.531	0.535	0.608	0.606	0.609
Medical studies and COVID-19 information	8.7%	6%	0.098	0.096	0.1	0.129	0.126	0.133
Loss and consolation	0.9%	1%	0.091	0.084	0.099	0.129	0.12	0.137
Account promotion	1%	1.2%	0.435	0.431	0.44	0.509	0.504	0.514
Picture challenges	1.5%	2.1%	0.474	0.469	0.478	0.53	0.526	0.535

**Table 3:** The conditional probability of a tweet belonging to a topic given that it belongs to the topic of politics.

Given (A):	Politics	
Subtopic (A):	US	UK
Public health and social values	0.155	0.151
Day-to-day life	0.175	0.216
Food	0.139	0.107
Professional achievements	0.075	0.120
Medical studies and COVID-19 information	0.049	0.041

**Table 4:** Correlation between the pandemic development and emotions.

Emotion		Virus Reproduction Rate		∆Death	
		US	UK	US	UK
Fear	Correlation	0.370	0.456		
	Significance	*p* = 0.012	*p* = 0.002		
	Lag	+1 week	+2 weeks		
Anger	Correlation		0.315	0.319	−0.485
	Significance		*p* = 0.033	*p* = 0.032	*p* = 0.001
	Lag		No lag	No lag	+4 weeks

## Data Availability

The data presented in this study are available on request from the corresponding author. The data are not publicly available due to ethical considerations.

## Appendix A

**Table A1:** List of professions and specializations of HCP points of interest.

Professions	Specializations
Anesthesiologist Assistant, Cardiovascular Technologist, Dialysis Technician, Emergency Medical Technician, Flight Nurse, Medical Laboratory Technician, Midwife, Nurse Anesthetist, Nurse Practitioner, Paramedic, Pharmacist, Phlebotomist, Physical Therapist, Physician Assistant, Radiation Therapist, Respiratory Therapist	Anesthesiology, Critical Care, Dermatology, Dermatopathology, Emergency Medicine, Family Medicine, Gynecology, Hepatology, Immunology, Internal Medicine, Neurology, Obstetrics, OB/GYN, Oncology, Ophthalmology, Pathology, Pediatrics, Pharmacy, Psychiatry, Radiology, Surgery, Urology, General Practitioner

**Table A2:** Mean values emotions for US and UK HCPs pre- and mid-pandemic.

	US	UK	*p*	US	UK	*p*
	Mean (*SD*) 2019	Mean (*SD*) 2019		Mean (*SD*) 2020	Mean (*SD*) 2020	
Fear	0.187 (0.004)	0.174 (0.007)	*p* < 0.001	0.196 (0.007)	0.181 (0.008)	*p* < 0.001
Anger	0.052 (0.001)	0.045 (0.001)	*p* < 0.001	0.053 (0.002)	0.047 (0.002)	*p* < 0.001
Sadness	0.101 (0.003)	0.085 (0.003)	*p* < 0.001	0.106 (0.003)	0.092 (0.003)	*p* < 0.001
Joy	0.373 (0.007)	0.408 (0.008)	*p* < 0.001	0.355 (0.007)	0.389 (0.01)	*p* < 0.001
Surprise	0.262 (0.005)	0.27 (0.005)	*p* < 0.001	0.265 (0.007)	0.27 (0.005)	*p* < 0.001
Disgust	0.024 (0.001)	0.019 (0.001)	*p* < 0.001	0.025 (0.001)	0.02 (0.001)	*p* < 0.001

**Table A3:** Mean values of emotions pre- and mid-pandemic by US and UK HCPs.

	US			UK		
	Mean (*SD*) 2019	Mean (*SD*) 2020	*p*	Mean (*SD*) 2019	Mean (*SD*) 2020	*p*
Fear	0.187 (0.004)	0.196 (0.007)	*p* < 0.001	0.174 (0.007)	0.181 (0.008)	*p* < 0.001
Anger	0.052 (0.001)	0.053 (0.002)	*p* = 0.002	0.045 (0.001)	0.047 (0.002)	*p* < 0.001
Sadness	0.101 (0.003)	0.106 (0.003)	*p* < 0.001	0.085 (0.003)	0.092 (0.003)	*p* < 0.001
Joy	0.373 (0.007)	0.355 (0.007)	*p* < 0.001	0.408 (0.008)	0.389 (0.01)	*p* < 0.001
Surprise	0.262 (0.005)	0.265 (0.007)	*p* < 0.001	0.27 (0.005)	0.27 (0.005)	*p* = 0.03
Disgust	0.024 (0.001)	0.025 (0.001)	*p* = 0.205	0.019 (0.001)	0.02 (0.001)	*p* = 0.001
